# A comparison of multi-ligament reconstruction and isolated anterior cruciate ligament reconstruction at one year follow-up: results from the Danish Knee Ligament Reconstruction Registry

**DOI:** 10.1186/s40634-022-00473-z

**Published:** 2022-04-07

**Authors:** Torsten Grønbech Nielsen, Ole Gade Sørensen, Martin Lind

**Affiliations:** Division of Sports Trauma, Orthopaedic Department, Aarhus University Hospital, Palle Juul-Jensens Boulevard 99, 8200 Aarhus N, Denmark

**Keywords:** Multiligament knee injury, Registry, Anterior cruciate ligament, Patient reported outcome

## Abstract

**Introduction:**

The Danish Knee Ligament Reconstruction Registry (DKRR) has monitored the outcomes of surgeries for multi-ligament knee injuries (MLKI) since 2005. This study aimed to compare the subjective clinical outcomes of patients who had undergone surgery after MLKI with those of patients who had received isolated anterior cruciate ligament (ACL) reconstruction.

**Materials and methods:**

This study used patient-reported outcome scores at 1-year follow-up as the primary outcome and contains the outcome data of knee ligament surgeries retrieved from the DKRR. Clinical subjective outcomes and knee function were evaluated with Knee Injury and Osteoarthritis Outcome Scores (KOOS) and Tegner Activity Scale (Tegner) scores. Demographic differences were examined using the Student’s t-test and the chi-square test. Multiple linear regression was used to analyse the data and adjust for potentially confounding factors. *P*-values < 0.05 were considered to be statistically significant.

**Results:**

A total of 31,686 knee ligament surgeries were registered in the DKRR between 2005 and 2017, resulting in 1,160 multi-ligament patients and 28,843 isolated ACL patients. The mean age of the MLKI group was significantly higher than that of the isolated ACL group (33.2 years [95% CI 32.5–33.9] vs. 28.3 years [95% CI 28.1–28.4]).

The adjusted KOOS Sport and Quality of Life (QoL) sub-scores and Tegner scores of the MLKI group significantly improved from the baseline to the 1-year follow-up (16.7 points [95%CI 12.8;20.6], 12.6 points [95%CI 9.6;15.6] and 1.76 points [95%CI 1.43;2.08], respectively). The KOOS Sport and QoL sub-scores of the isolated ACL group were significant and increased more than those of the MLKI group. No differences in the Tegner scores were observed.

**Conclusions:**

Surgical reconstruction after multi-ligament knee injury resulted in significant subjective outcome improvements at 1- year follow-up. The KOOS Sport and QoL sub-scores of the isolated ACL group significantly increased compared to those of the MLKI group.

## Introduction

Multi-ligament knee injuries (MLKI) are uncommon, with a reported occurrence of less than 0.02% of all orthopaedic injuries [5, 12, 19]. Therefore, many studies reporting outcomes after MLKI and knee dislocation include relatively few patients. Because of the high diversity of ligament injuries and supplemental intra- and extra-articular lesions in knee dislocation, high volume patient studies are required to identify optimal treatments. However, few studies have represented patient-reported outcomes with large cohorts of more than 100 patients [11, 13, 15, 18].

Data from regional and national registries represent a possible source of high volume MLKI patient cohorts. A 2014 study from Finland that collected data from 837 patients [23] was the first population-based registry study to present the incidence of acute knee dislocation. Wilson et al. [24] followed shortly after with a registry-based study on knee dislocation epidemiology. Notably, neither of these studies used patient-reported outcome scores.

The Danish Knee Ligament Reconstruction Registry (DKRR) has monitored ligament knee injuries since 2005. The aim of the study was to compare subjective patient-reported outcomes after MLKI with outcomes after isolated anterior cruciate ligament (ACL) reconstruction. Thus, it used data from the DKRR to evaluate patient outcomes after MLKI, resulting in a cohort of 1,160 MLKI patients. To the best of our knowledge, this is the largest study of patients presenting subjective clinical outcomes after multi-ligament reconstruction surgery.

### Purpose

To compare the subjective clinical outcomes of patients who had undergone surgery after MLKI with those of patients who had received isolated ACL reconstruction.

## Materials and methods

The nationwide DKRR was established in 2005 [14]. The DKKR is a prospective web-based clinical quality database that contains data on patients’ characteristics and surgical details after knee ligament reconstruction. The database registers both ligament revision surgeries and reconstructions of isolated cruciate ligaments, collateral ligaments and multi-ligaments. It is mandatory for surgeons to register all surgical details in the DKKR, and the data are collected pre-surgery, intra-operatively and at 1-year follow-up. Depending on their ligament reconstructions, the patients in this study were included to either the MLKI group or the isolated ACL group.

### Patient-reported outcome scores

In this study, the patients independently evaluated their knee condition before surgery and at 1-year follow-up using self-assessment questionnaires, namely the Tegner Activity Scale (Tegner) [3] and the Knee Injury and Osteoarthritis Outcome Score (KOOS) [20]. The KOOS score is divided into 5 sub-scores: Symptoms, Pain, Activity of Daily Living (ADL), Sport and Recreation (Sport) and Quality of Life (QoL). Only the KOOS Sport and QoL sub-scores are sensitive to ACL patients [8], this study used these sub-scores and the Tegner score for further analysis.

### Patient characteristics

Patient characteristics in terms of age, gender, injury mechanism, time from injury to surgery, meniscal lesions and cartilage lesions were retrieved from the DKRR. The MLKI patients were subdivided according to Schenck’s knee dislocation classification [22] and isolated ACL. The Schenck’s knee dislocation classification are used to present the knee surgeries which have been performed in the MLKI group. Schenck divided Knee dislocation (KD) injuries in 5 subgroups. KD I was torn of single cruciate ligament and either one or both of collaterals. KD II was injuries of both cruciate ligaments and intact collaterals. KD III is injury to both cruciate ligaments and either medial or lateral collateral. KD IV was injuries to both cruciate ligaments and collaterals. KD-V was dislocation and a knee fracture. Sub-group KD III was split up to at medial side (KD IIIM) and a lateral side (KD IIIL) [22]. Schenck’s knee dislocation classification was not used for subgroup analysis or outcomes.

### Approvals

This study was approved by the Danish Board of Health and the Danish Data Protection Agency (approval no.: 1–16-02–728-18). National clinical registry studies do not require local ethical committee approval in Denmark.

### Statistics

The data were normally distributed, and the Student’s t-test and the chi-square test were used to calculate the demographic differences between the MLKI and isolated ACL groups. Multiple linear regression was used to analyse the data and adjust for potentially confounding factors such as age, gender, meniscus lesions and cartilage lesions. Responsiveness was calculated as the ratio of the number of patients who returned outcome scores to the number of patients who had undergone surgery. The results are presented as means with 95% confidence intervals (CI). *P*-values < 0.05 were considered to be statistically significant. Statistical analysis was performed using Stata Software Version 17 (StataCorp, College Station, Texas 77,845, USA).

## Results

A total of 31,686 knee ligament surgeries were registered in the DKRR between 2005 and 2017. Isolated PCL (313 patients), revisions (1,282 patients) and patients with acute surgery (88 patients) were excluded from this study, resulting in 1,160 multi-ligaments representing 3.9% of all primary reconstructions. In contrast, 28,843 patients had received ACL reconstruction (96.1%). Statistically significant differences between the MLKI group and the isolated ACL group were observed for most demographic parameters. Table 1 presents the demographic data used in this study.

**Table 1:** Patient demographics

	**Multi-ligament**	**Isolated ACL**	***p*-values**
Patients, n (%)	1,160 (3.9)	28,843 (96.1)	
Age, years (mean [95%CI])	33.2 (32.5–33.9)	28.3 (28.1–28.4)	< 0.001^a^
Gender male: female (ratio)	70:30	60:40	< 0.001^b^
Meniscus injury, n (%)	357 (31)	12,427 (43)	< 0.001 ^b^
Cartilage injury, n (%)	514 (44)	13,753 (48)	0.027 ^b^
Time from injury to surgery, month (mean [95%CI])	24.1 (21.3–27.0)	20.5 (20.1–21.0)	0.005 ^a^
**Knee dislocation classification (Schenck)**			
- KD I, n (%)	903 (78)		
- KD II, n (%)	86 (7)		
- KD IIIM, n (%)	71 (6)		
- KD IIIL, n (%)	88 (8)		
- KD IV, n (%)	12 (1)		
**Injury mechanism**			
- ADL, n (%)	197 (17)	2,347 (8)	< 0.001 ^b^
- Traffic, n (%)	216 (19)	812 (3)	
- Work, n (%)	64 (6)	732 (3)	
- Sport—pivoting, n (%)	335 (29)	16,717 (58)	
- Sport—non-pivoting, n (%)	304 (26)	7,126 (25)	
- Unknown, n (%)	44 (4)	1,109 (4)	
**Patient reported outcome scores (Baseline)**			
**KOOS**	*n* = *371 (32%)*	*n* = *10,057 (35%)*	
- Symptoms (mean [95%CI])	68.3 (66.6–70.1)	71.1 (70.8–71.4)	< 0.001 ^a^
- Pain (mean [95%CI])	65.3 (63.2–67.4)	71.3 (70.9–71.6)	< 0.001 ^a^
- ADL (mean [95%CI])	69.1 (66.8–71.3)	78.9 (78.5–79.2)	< 0.001 ^a^
- Sport (mean [95%CI])	26.7 (24.0–29.3)	38.0 (37.5–38.5)	< 0.001 ^a^
- QOL (mean [95%CI])	32.9 (31.1–34.7)	39.0 (38.7–39.3)	< 0.001 ^a^
**Tegner** (mean [95%CI])	2.3 (2.1–2.5)	3.1 (3.0–3.1)	< 0.001 ^a^

Data presented as the number of individuals (n (%) and mean (95%CI). Acute: < 3 weeks, chronic: > 3 weeks, *KD* Knee dislocation, *ADL* Activity of Daily Living, Sport – pivoting: Football and handball, Sport – non-pivoting: All other sports. ^a^Student’s t-test. ^b^Chi-square test

Tables 2, 3 and 4 display the crude and adjusted improvements in the KOOS Sport and QoL sub-scores and the Tegner scores from the baseline measure to the 1-year follow-up. The overall completeness of subjective scores at 1-year follow-up was 37% and 31% for MLKI and ACL patients, respectively.

**Table 2:** Crude and adjusted differences in the KOOS Sport sub-scores of the MLKI group between baseline and 1-year follow-up and the impact of risk factors (gender, age, meniscus and cartilage injury)

	**Crude (mean[95%CI])** *n* = *5093*	**Adjusted (mean[95%CI])** *n* = *5085*	***p*-values**
***Mean difference (Isolated ACL [controls])***	*21.49 (20.73;22.30)*	*21.75 (20.20;23.29)*	< *0.001*
**Mean difference (MLKI)**	16.69 (12.88;20.50)	16.67 (12.79;20.59)	< *0.001*
**Mean difference (MLKI vs Isolated ACL)**	4.81 (0.95;8.66)	5.06 (1.17;8.94)	*0.01*
**Gender (female)**		-0.50 (-2.02;1.02)	0.52
**Age (year)**		0.07 (0.001;0.15)	*0.05*
**Meniscus injury (yes)**		1.98 (0.44;3.53)	*0.01*
**Cartilage injury (yes)**		-1.26 (-2.81;0.30)	0.11

Regression model adjusted for gender, age, meniscus and cartilage injury. Data presented as mean (95%CI). *p*-values < 0.05 indicates if the variable influence on the adjusted mean difference

**Table 3:** Crude and adjusted differences in the KOOS QoL sub-scores of the MLKI group between baseline and 1-year follow-up and the impact of risk factors (gender, age, meniscus and cartilage injury)

	**Crude (mean[95%CI])** *n* = *5,093*	**Adjusted (mean[95%CI])** *n* = *5,085*	***p*-values**
***Mean difference (Isolated ACL [controls])***	*18.03 (17.43;18.62)*	*17.43 (16.24;18.62)*	< *0.001*
**Mean difference (MLKI)**	13.51 (10.72;16.31)	12.61 (9.61;15.61)	< *0.001*
**Mean difference (MLKI vs Isolated ACL)**	4.51 (1.54;7.48)	4.82 (1.83;7.81)	*0.002*
**Gender (female)**		0.32 (-0.85;1.49)	0.53
**Age (year)**		0.11 (0.05;0.16)	< *0.001*
**Meniscus injury (yes)**		1.59 (0.40;2.79)	*0.009*
**Cartilage injury (yes)**		0.01 (-1.19;1.21)	0.98

Regression model adjusted for gender, age, meniscus and cartilage injury. Data presented as mean (95%CI). *p*-values < 0.05 indicates if the variable influence on the adjusted mean difference

**Table 4:** Crude and adjusted differences in the Tegner scores of the MLKI group between baseline and 1-year follow-up and the impact of risk factors (gender, age, meniscus and cartilage injury)

	**Crude (mean[95%CI])** *n* = *5,088*	**Adjusted (mean[95%CI])** *n* = *5,080*	***p*-values**
***Mean difference (Isolated ACL [controls])***	*1.83 (1.77;1.89)*	*1.89 (1.76;2.02)*	< *0.001*
**Mean difference (MLKI)**	1.52 (1.17;1.88)	1.76 (1.43;2.08)	< *0.001*
**Mean difference (MLKI vs Isolated ACL)**	0.31 (-0.02;0.63)	0.13(-0.19;0.46)	0.42
**Gender (female)**		-0.16 (-0.29;-0.04)	*0.01*
**Age (year)**		-0.04 (-0.04;-0,03)	< *0.001*
**Meniscus injury (yes)**		-0.02 (-0.15;0.11)	0.79
**Cartilage injury (yes)**		-0.09 (-0.22;0.04)	0.18

Regression model adjusted for gender, age, meniscus and cartilage injury. Data presented as mean (95%CI). *p*-values < 0.05 indicates if the variable influence on the adjusted mean difference

The MLKI group significantly improved their KOOS Sport sub-scores (16.67 points [95%CI 12.79;20.59]). Those with meniscus injuries significantly improved their mean scores (1.98 points [95%CI 0.44;3.538]). Gender, age and cartilage injury did not significantly influence the Sport sub-scores. Higher age had a tendency to obtain higher sub-scores (*p* = 0.05). The isolated ACL group significantly improved their Sport sub-scores (21.75 points [95%CI 20.20;23.29]) compared to the MLKI group (Table 2).

Notably, a significant improvement was seen in the KOOS QoL sub-scores (12.61 points [95%CI 9.61;15.61]). In particular, higher age and meniscus injury led to a significant increase in the QoL sub-scores (0.11 points/year [95%CI 0.05;0.16] and 1.59 points [95%CI 0.40;2.79], respectively). No significant improvements in relation to gender or cartilage injury were observed. Compared to the MLKI group, the condition of the isolated ACL group significantly improved from the baseline to the 1-year follow-up (17.43 points [95%CI 16.24;18.62]) (Table 3).

Furthermore, the Tegner scores of the MLKI group significantly improved (1.76 points [95%CI 1.43;2.08]). Female gender and higher age significantly decreased Tegner scores (0.16 points [95%CI 0.04;0.29]; 0.04 points/year [95%CI 0.03;0.04], respectively). Meniscus injury and cartilage lesions did not significantly influence the differences in the Tegner scores (0.79 and 0.18, respectively). The condition of the isolated ACL group clearly improved (1.89 points [95%CI 1.76;2.02]) from the baseline to the 1-year follow-up (Table 4).

## Discussion

The most important finding in this study was that the patients in the MLKI group benefited from the surgical reconstruction treatment and reported significant improvements in their KOOS Sport and QoL sub-scores and Tegner scores. However, these improvements were lower than those of the isolated ACL group.

In this study, age significantly influenced the improvements in the QoL sub-scores and Tegner scores. Increased age led to a decrease in the Tegner scores and an increase in the Sport and QoL sub-scores. One other study used age as a comparator to analyse the data of MLKI patients. In contrast to the present study, Levy et al. [13] found that patients below 30 years of age had significantly higher patient-reported outcome scores than those above 30 years of age. Levy et al. used IKDC and Lysholm scores and these scores are predominantly activity focused whereas KOOS scores are symptom focused. This can explain the finding of higher scores for higher age as the high age group have a reduced activity level which likely results in reduced symptoms and thereby higher KOOS-scores. Also baseline-level differences between the present and the Levy study were not taken into account. Notably, younger patients are more likely to have higher activity levels than their older counterparts. Thus, even though this study observed that increased age led to significant improvements in the KOOS sub-scores, age does not seem to be clinically relevant.

Our results showed that the female patients had lower Tegner scores than the male patients. The current study is the first to present this comparison. As with age, the difference between men and women was small and might not be clinically relevant.

This study subdivided patients in the MLKI group according to Schenck’s knee dislocation classification (Table 1). A Schenck subgroup outcome would have been beneficial to analyse the subgroup differences in relation to two, three or four ligament reconstruction. Due to low patient-recorded outcome completeness, this analysis was not possible. Billieres et al. [1] found that KD-I patients showed impaired subjective outcomes compared to KD-II and KD-III patients using Tegner, Lyshom and IKDC scores. Due to the low number of patients, differences of 10–12 points were not statistically significant. Another study by Engebretsen [4] found that KD-IV patients had impaired subjective outcomes compared to KD-II and KD-III patients using Tegner, Lyshom and IKDC scores. Only IKDC improvement was significant: 68 in the KD-II and KD-III group and 51 in the KD-IV group. The groups contained 73 and 10 participants, respectively. Ranger et al. [17] provided the same results as Billieres and Engebretsen [1, 4]. Differences between KD-IV and KD-II/KD-III patients were observed in relation to their IKDC, Tegner and Lysholm scores, but due to the low completeness of the IKDC and Tegner scores, only the Lysholm score significantly improved in the KD-II/KD-III group compared to the KD-IV group at the 24-month follow-up.

Regarding concomitant knee lesions, 31% of the patients in the MLKI group had at least one meniscal lesion, whereas 44% had cartilage lesions. In the MLKI group, meniscus injury had a surprisingly significant positive influence on the KOOS Sport and QoL sub-scores, whereas cartilage injury did not influence these sub-scores. King et al. [10] observed an inferior outcome in patients with cartilage injuries or bilateral meniscal tears. They reported cartilage defects in 40% and meniscal lesions in 55% of the patients. Moatshe el al. [15] found meniscal lesions in 37% of the cases. Most patients in the present study underwent surgery in a delayed setup. Thus, the lower percentage of meniscal lesions in this study compared to other studies could be the result of meniscal healing in the period from accident to surgery. In contrast, a recent systematic review by Kim et al. [9] associated prolonged time to surgery with an increased risk of both meniscal injury and cartilage lesions in MLKI patients. In line with the present study, Moatshe et al. [15] located cartilage defects in 20% of patients undergoing surgery in an acute setup and 47.7% of patients in a delayed setup.

### Limitations

First, the overall degree of patient responsiveness at the 1-year follow-up was 37%, which could result in biased patient-reported outcome scores. Nonetheless, Rahr-Wagner et al. [16] validated the DKRR and found no differences in the KOOS and Tegner scores of responders and non-responders, which indicates that the high percentage of non-responders in the present study did not create biased scores.

Second, the DKRR only presents outcome scores based on the 1-year follow-up to a knee ligament reconstruction procedure. This may represent a time point at which full recovery and rehabilitation have not been achieved because of possible improvements in patient performance between the 1- and 2-year period following surgery. However, regarding knee ligament reconstructions, data from the Swedish ACL registry [21] show no significant improvements in the outcome scores from the 2-year follow-up compared to those from the 1-year follow-up. This might not be the same for MLKI patients. MLKI patients might need a longer rehabilitation period due to reach a more fully recovery compared to ACL patients.

Third, this study did not assess the crucial risk of vascular injury and neurological complications after MLKI, which has been reported in several studies [2, 6, 7, 23]. Because the DKRR contains no data on these complications, it was impossible for this study to quantify them.

## Conclusions

This study found that surgical reconstruction after multi-ligament knee injury resulted in significant subjective outcome improvements at the 1-year follow-up. Patients with isolated ACL reconstruction had significantly higher KOOS Sport and QoL sub-scores than MLKI patients. Further studies with higher patient-reported outcome completeness and longer follow-up periods are needed.
